# Tocilizumab in combination with corticosteroids: potential for managing cancer cachexia with systemic hyperinflammation

**DOI:** 10.3389/fimmu.2024.1477310

**Published:** 2024-12-05

**Authors:** Ping Chen, Dingyi Wang, Zhouwei Zhan, Ling Chen, Yu Chen

**Affiliations:** Department of Medical Oncology, Clinical Oncology School of Fujian Medical University, Fujian Cancer Hospital, Fuzhou, Fujian, China

**Keywords:** cachexia, cancer, inflammation, IL-6 receptor, tocilizumab

## Abstract

**Background:**

Cachexia is a leading cause of death among individuals with advanced cancer, yet effective pharmacological treatments are lacking. In this single-center retrospective study, we aimed to investigate the efficacy and safety of tocilizumab for the treatment of cancer cachexia accompanied by systemic hyperinflammation.

**Methods:**

Data were collected from 20 patients treated with tocilizumab and a control group of 20 patients matched for age, sex, and comorbidities. Both groups received corticosteroids. In the tocilizumab treatment group, patients received a single dose of tocilizumab (8 mg/kg, maximum 800 mg) in combination with corticosteroids. Weight, body mass index, liver metastasis, Eastern Cooperative Oncology Group score, patient-generated subjective global assessments, the Anorexia/Cachexia Subscale of the Functional Assessment of Anorexia/Cachexia Therapy, handgrip strength, neutrophil-to-lymphocyte ratio, and the C-reactive protein, hemoglobin, prealbumin, and albumin levels were recorded in both groups.

**Results:**

Tocilizumab treatment favorably influenced the levels of patient biomarkers (p<0.05), ameliorated systemic inflammation, and demonstrated enhanced clinical short-term efficacy compared to the control group, including rates of symptomatic relief (60% vs. 20%, p = 0.024), improvement of serum PAB and ALB (70% vs. 25%, p = 0.004), weight gain >2% (45% vs. 15%, p = 0.038), and improvement of grip strength and 6-m walk speed (p<0.05). Treatment with tocilizumab was generally safe, with no observed increase in infection rates (10% vs. 15%, p = 0.633) or intensive care unit admissions (10% vs. 25%, p = 0.405), and was more favorable for restarting antitumor therapy (70% vs. 35%, p = 0.027).

**Conclusions:**

Tocilizumab, in combination with corticosteroids, is favorable for alleviating cancer cachexia with systemic hyperinflammation, despite the small sample size. Thus, this combination holds great potential as a novel strategy for treating cancer cachexia with systemic hyperinflammation.

## 1 Introduction

Cancer cachexia is a common multifactorial syndrome in patients with advanced tumors. It is characterized by loss of skeletal muscle mass, poor nutritional status, and elevated levels of inflammation, posing a serious threat to patient prognosis, survival, and overall quality of life (1). Current treatments for cachexia remain restricted to the use of glucocorticoids, progestins, and other appetite-enhancing medications, complemented by essential nutritional support (2). However, the overall efficacy of this approach is unsatisfactory. A significant proportion of patients fail to respond to treatment and progress to the refractory stage of cachexia, with complications such as electrolyte disorders and multiorgan failure, ultimately causing death. Cancer cachexia is highly associated with cancers of the pancreas, esophagus, stomach, lung, liver, and bowel, particularly at the end stage of life. Notably, approximately half of annual cancer-related deaths worldwide (approximately 8.2 million) are attributed to cancer cachexia (3). Therefore, effective treatments are urgently needed.

Cachexia is instigated by a combination of diminished food intake and alterations in body metabolism, where hyperinflammation and hypermetabolism play crucial roles in disease progression (1, 4). Tumors induce heightened inflammation and release various pro-inflammatory cytokines, eicosanoids, and other factors with tissue-specific effects that promote catabolism in target organs, such as skeletal muscle, adipose, and myocardium. These factors contribute to the outward signs of anorexia and fatigue in patients through complex neuroendocrine regulatory mechanisms (5, 6). Interleukin-6 (IL-6) stands out as a crucial factor in this process. As a pleiotropic cytokine, IL-6 is associated with muscle atrophy, tumorigenesis, and hepatic acute phase protein (such as C-reactive protein) production, as well as various symptoms, such as anorexia, reduced serum albumin, reduced hemoglobin (Hb), and reduced body mass index (BMI) (7, 8). Therefore, inhibiting IL-6 while ensuring energy intake in patients with cachexia holds promise for halting or reversing the hypermetabolic state and may be an effective treatment for cachexia.

Tocilizumab (TCZ) is an anti-human IL-6 receptor monoclonal antibody that rapidly normalizes acute-phase protein levels and effectively inhibits the development of systemic inflammation (9). TCZ has been widely utilized in the treatment of rheumatic immune diseases, including rheumatoid arthritis, giant cell arteritis, and adult-onset Still’s disease (10). In 2013, Ando et al. reported a case of a patient with malignant lung cancer and cachexia treated with TCZ, resulting in a prolonged survival of 9 months without antitumor therapy. During this period, a significant improvement in symptoms and rapid recovery of all indicators were observed (11). Similar cases were subsequently reported, albeit limited to isolated cases (12).

This was an exploratory study aimed to investigate the efficacy and safety of TCZ for the treatment of cancer cachexia with systemic hyperinflammation. We compared symptoms and laboratory parameters in patients who received TCZ treatment to those who did not. We hypothesized that TCZ would play a beneficial therapeutic role in cachexia associated with systemic hyperinflammation.

## 2 Materials and methods

This single-center retrospective controlled study was conducted in the Department of Medical Oncology, Fujian Cancer Hospital, Fuzhou, Fujian, China.

### 2.1 Study population

Data from patients with advanced cancer accompanied by cachexia between January 10, 2023, and February 1, 2024 were retrospectively analyzed. Follow-up data were collected until May 1, 2024. The inclusion criteria were as follows: rapid weight loss (involuntary) combined with systemic inflammation over a short period and a diagnosis of cachexia confirmed by a clinician according to the Fearon criteria (1). For instance, these criteria include weight loss >5% over the past 6 months (excluding simple starvation), or a BMI <20 kg/m^2^ with any amount of weight loss >2%, or appendicular skeletal muscle index consistent with sarcopenia (males <7·26 kg/m²; females <5·45 kg/m²)* and any degree of weight loss >2%. The exclusion criteria were previous autoimmune disease, administration of immune checkpoint inhibitor therapy within the past month, history of erythrocyte or albumin (ALB) infusion, no inflammation, clinically confirmed infection, contraindication to glucocorticoid therapy, comorbidities involving substantial pleural or abdominal fluid, and a follow-up duration of <1 month. All patients were unable to receive antitumor therapy because of their poor general condition.

All patients underwent pathogen examination, including blood, sputum, and stool cultures. Additionally, radiological assessments, such as color Doppler ultrasound or computed tomography, were conducted to exclude any evident infections. Clinical characteristics, including sex, age, BMI, weight, type of cancer, presence of liver metastasis, Eastern Cooperative Oncology Group (ECOG), patient-generated subjective global assessment (PG-SGA), the Anorexia/Cachexia Subscale of the Functional Assessment of Anorexia/Cachexia Therapy (FAACT-A/CS), handgrip strength, and 6-m walk speed were collected. The PG-SGA serves as a rapid and effective evaluation tool for identifying and predicting malnutrition in hospitalized oncology patients (13). The FAACT-A/CS is a patient self-reported assessment scale. It is increasingly utilized in clinical studies related to cachexia to assess appetite and the impact of anorexia/cachexia on patients’ quality of life. The FAACT-A/CS is scored according to the FACIT manual. It comprises 12 items rated on a five-point Likert scale (0 = not at all, 1 = a little, 2 = somewhat, 3 = quite a bit, 4 = very much). The total score ranges from 0 to 48, with lower scores indicating poorer appetite. The optimal threshold value validated by the University of Amsterdam in the Netherlands is 37 points (14). Grip strength and 6-m walk speed are important tools published by the Asian Working Group for Sarcopenia for the assessment of muscle function (15). Laboratory investigations included the serum neutrophil-to-lymphocyte ratio (NLR), C-reactive protein (CRP) level, Hb level, prealbumin (PAB) level, and ALB level.

### 2.2 Trial design

Patients were divided into the TCZ and characteristics-matched control groups based on prior medication. All patients underwent standard glucocorticoid intravenous therapy (methylated prednisolone 20 mg once daily) (16) and received adequate parenteral nutritional support [25–30 kcal/(kg*d)]. The TCZ treatment group received an additional single intravenous dose of TCZ (8 mg/kg, maximum 800 mg). The patients were observed in the hospital for at least 1 week following treatment. The decision to discharge each patient was made based on their individual circumstances after 1 week. Upon discharge, the glucocorticoids were switched to oral administration at an equivalent dose.

A comprehensive assessment of patient parameters, including symptoms, inflammation, nutritional status, co-infections, intensive care unit (ICU) admissions, re-antitumor therapy, and tumor response, was performed. Our data collection was facilitated by routine hematological examinations conducted 1–2 times/week and nutritional screening once a week. The criteria for relief of clinical symptoms were no fever for >72 h and FAACT-A/CS >37 points. Improved serum PAB and ALB levels were defined as serum PAB levels >180 mg/L and ALB levels >35 mg/L. Complicated infections were identified by clear evidence of pathogenesis or imaging. Re-antitumor therapy refered to the resumption of antitumor treatments, including chemotherapy, immunotherapy, or targeted therapy, following stabilization or clinical improvement after the initial treatment with TCZ or corticosteroids. Tumor response was assessed according to the RECIST 1.1 criteria. Patient follow-up was conducted for 1 month at least during outpatient and inpatient visits. All parameters were obtained from our central laboratory.

### 2.3 Statistical analysis

Statistical analysis was conducted using IBM SPSS Statistics 25 (IBM Corp., Armonk, NY, USA) and GraphPad Prism 8 (GraphPad Software Inc., San Diego, CA, USA). *T*-tests were used for parametric data, whereas Mann–Whitney U tests were used for non-parametric data. Data are presented as means and standard deviations, or medians with ranges. Categorical data were analyzed using Pearson’s chi-squared test. Kaplan–Meier curves were used to compare the time to an event between the two groups. The level of significance was set at p<0.05.

### 2.4 Ethical approval and consent to participate

This study was approved by the Ethics Committee of Fujian Provincial Cancer Hospital (No. K2023-234-01) and conducted in accordance with the principles of the Declaration of Helsinki. Written informed consent was obtained from all patients for the use of their medical records for research purposes.

## 3 Results

### 3.1 Patient characterization

**Figure 1:**
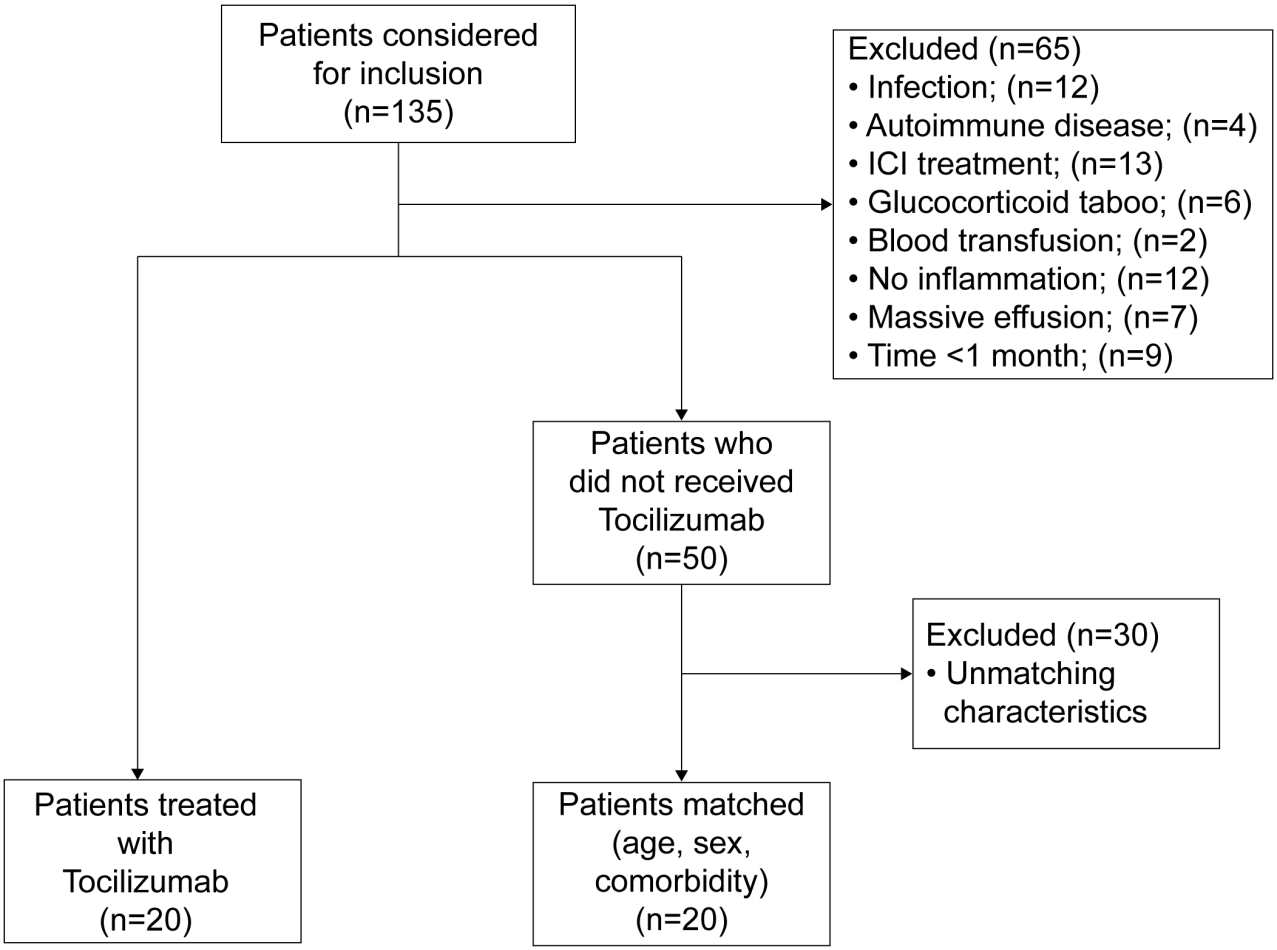
The flow of patients from analysis to inclusion and matching.

A total of 135 individuals were diagnosed with cachexia between January 10, 2023, and February 1, 2024. 65 individuals were excluded based on predefined criteria. Among these, 9 lost contact and were not treated with TCZ, while the remaining individuals survived for more than 1 month. Of the remaining individuals, 20 patients opted for treatment with TCZ based on physician recommendations and were included in the TCZ group. The remaining 50 patients were analyzed for age, sex, and comorbidities, and 20 matched controls were included. The inclusion criteria and patient flow are shown in **Figure 1**.

Each group consisted of 10 males and 10 females, for a total of 20 males and 20 females across both the TCZ-treated and control groups. All participants had advanced, progressive cancer, with gastrointestinal cancer being the most prevalent underlying diagnosis (n = 15, 37.5%), followed by melanoma (n = 11, 27.5%), sarcoma (n = 6, 15%), genitourinary cancer (n = 5, 12.5%), and other cancers (n = 3, 7.5%). The age range of the participants in the TCZ and control groups was 60 (22–80) years and 59 (21–83) years, respectively. No significant differences were observed in sex, age, weight, BMI, presence of liver metastases, ECOG score, PG-SGA score, FAACT-A/CS score, handgrip strength, or 6-m walk speed between the two groups. Additionally, there were no significant differences in baseline laboratory biomarkers, including the NLR, CRP, HB, PAB, and ALB levels, between the two groups (**Table 1**).

**Table 1:** Baseline characteristics of participants from the TCZ-treated and control groups.

Parameters	TCZ-Treated	Control (TCZ-Untreated)	p-value
Sex, n (%)			1
Male	10 (50)	10 (50)	
Female	10 (50)	10 (50)	
Age, (years), Median (range)	60 (22–80)	59 (21–83)	0.989
Weight, Median (range)	56.5 (38–74)	55.6 (35–75)	0.678
BMI, Median (range)	22.6 (14.4–26)	22.0 (15.1–25.4)	0.820
Type of cancer, n (%)			0.951
Gastrointestinal cancer	8 (40)	7 (35)	
Melanoma	5 (25)	6 (30)	
Sarcoma	3 (15)	3 (15)	
Genitourinary cancer	3 (15)	2 (10)	
Other cancer	1 (5)	2 (10)	
Liver metastases, n (%)			0.749
Yes	9 (45)	8 (40)	
No	11 (65)	12 (60)	
ECOG score, n (%)			0.926
1	4 (20)	5 (25)	
2	12 (60)	11 (55)	
3	4 (20)	4 (20)	
PG-SGA score, Median (range)	12 (7–26)	14 (9–17)	0.445
FAACT-A/CS score, Median (range)	27 (17–36)	27.5 (18–36)	0.820
Handgrip strength (kg), Median (range)	21.9 (15.4–39.7)	21.2 (13.2–37.6)	0.883
6-m walk speed (m/s), Median (range)	0.9 (0.6–1.1)	0.9 (0.6–1.1)	0.565
NLR, Median (range)	7.9 (1.9–30)	6.4 (2.9–24.5)	0.678
CRP (mg/L), Median (range)	102 (48.2–200)	73.5 (42.4–197.6)	0.096
Hemoglobin (g/L), Median (range)	96 (63–139)	96 (62–146)	0.758
Prealbumin (mg/L), Median (range)	96.5 (15–230)	85 (35–155)	0.779
Albumin (g/L), Median (range)	29.8 (23.1–35)	30.1 (21.2–34)	0.779

BMI, body mass index; CRP, C-reactive protein; ECOG, Eastern Cooperative Oncology Group; ICU, intensive care unit; NLR, neutrophil-to-lymphocyte ratio; PG-SGA, patient-generated subjective global assessment; SD, standard deviation; TCZ: Tocilizumab.

### 3.2 Effect of TCZ on symptoms and laboratory parameters

**Figure 2:**
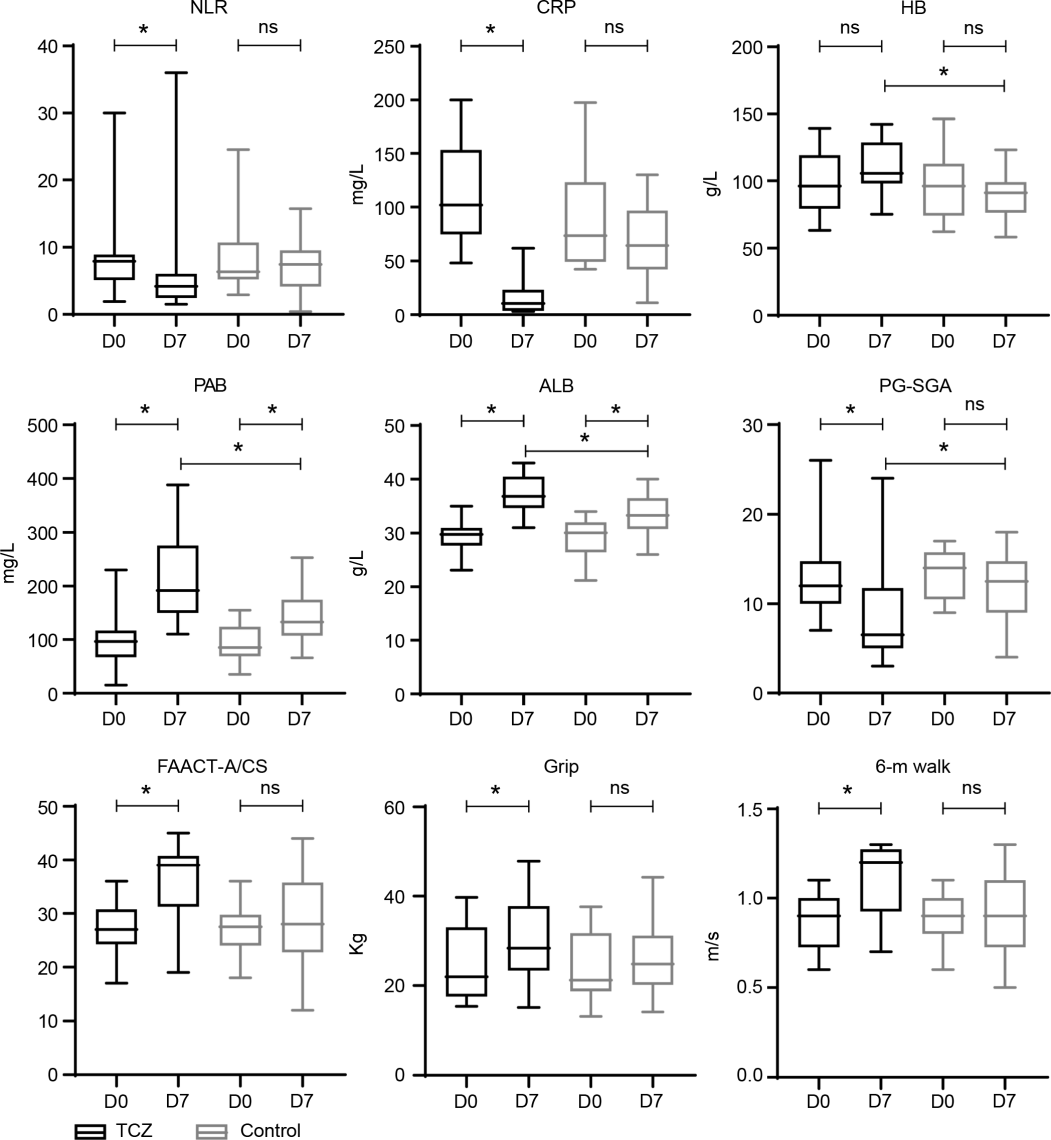
Laboratory parameters of TCZ-treated and control groups. D 0—baseline; D 7—day 7 after treatment; D 30—approximately 30 days from the baseline. * statistically significant; ALB, albumin; CRP, C-reactive protein; Hb, hemoglobin; NLR, neutrophil-lymphocyte ratio; ns, statistically non-significant; PAB, prealbumin.

**Figure 3:**
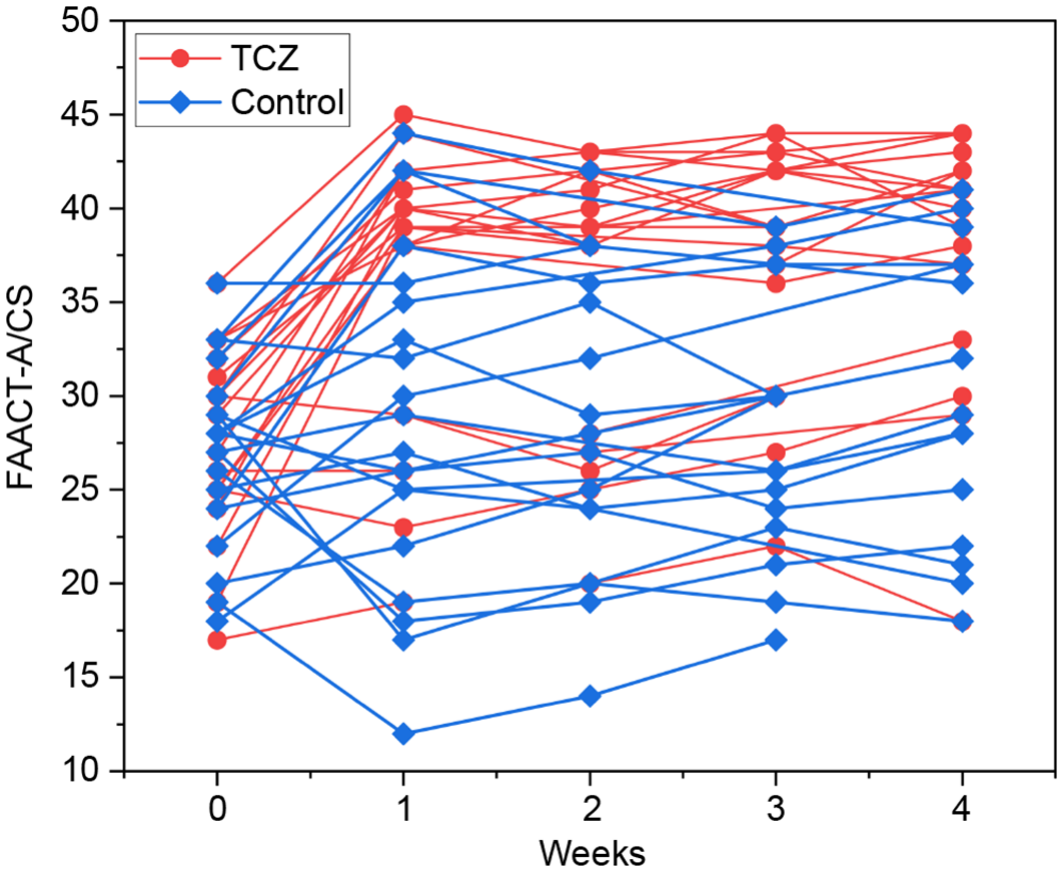
FAACT-A/CS change of patients within one month.

The patients in the TCZ group exhibited a higher rate of symptom relief 1 week after TCZ treatment than those in the control group (60% vs. 20%, p = 0.024) (**Table 2**). FAACT-A/CS scores significantly improved after 7 days of TCZ treatment compared to baseline scores [27 (17–36) vs. 39 (19–45), p = 0.001], while the control group showed no significant improvement [27.5 (18–36) vs. 28 (12–44), p = 0.498] (**Figure 2**). Changes in FAACT-A/CS scores during the follow-up in both groups are shown in **Figure 3**. TCZ treatment resulted in a significant decrease in NLR [7.9 (1.9–30) vs. 4.2 (1.5–36), p = 0.005] and CRP [102 (48.2–200) vs. 10.6 (3.1–62), p = 0.001] levels, along with a significant increase in serum PAB [96.5 (15–230) vs. 192 (110–388), p = 0.001] and ALB [29.8 (23.1–35) vs. 36.9 (31–43), p = 0.001] levels compared with pre-treatment values. No significant changes in NLR [6.4 (2.9–24.5) vs. 7.5 (0.4–15.7), p = 0.841] or CRP [73.5 (42.4–197.6) vs. 64.5 (11.1–130), p = 0.201] levels were observed in the control group pre- and post-treatment. Day 7 serum PAB [85 (35–155) vs. 132.5 (66–253), p = 0.002] and ALB [30.1 (21.2–34.0) vs. 33.3 (26–40), p = 0.003] levels were higher than those on Day 0, but still lower than those in the TCZ group (p<0.05). Hb [105 (75–142) vs. 91 (58–123), p = 0.002] levels were significantly higher in the TCZ-treated group compared to the control group on Day 7, while there were no significant changes in Hb levels before and after treatment in either group (**Figure 2**).

**Table 2:** Outcome measures after treatment.

Parameters	TCZ-Treated	Control (TCZ-Untreated)	p-value
Symptom relief within 1 week, n (%)			0.024
Yes	12 (60)	4 (20)	
No	8 (40)	16 (80)	
Weight, Median (range)^a^	57.7 (39.5–72)^ns^	55.7 (35.5–76)^ns^	0.495
Infection, n (%)^b^			0.633
Yes	2 (10)	3 (15)	
No	18 (90)	17 (85)	
Weight gain, n (%)^a^			0.185
Yes	15 (75)	11 (55)	
No	5 (25)	9 (45)	
Weight gain of more than 2%, n (%)^a^			0.038
Yes	9 (45)	3 (15)	
No	11 (55)	17 (85)	
Re-antitumor, n (%)^b^			0.027
Yes	14 (70)	7 (35)	
No	6 (30)	13 (65)	
ICU admission, n (%)^b^			0.405
Yes	2 (10)	5 (25)	
No	18 (90)	15 (75)	
Tumor response, n (%)^b^			0.439
Partial response	3 (15)	1 (5)	
Stable disease	6 (30)	9 (45)	
Progressive disease	11 (55)	10 (50)	

ICU, intensive care unit; ns, statistically non-significant compared to pre-treatment; a, approximately 30 days from the baseline; b, within 1 month; Re-antitumor, resumption of antitumor treatments after the initial treatment with tocilizumab or corticosteroids.

### 3.3 Impact of TCZ on nutritional status

**Figure 4:**
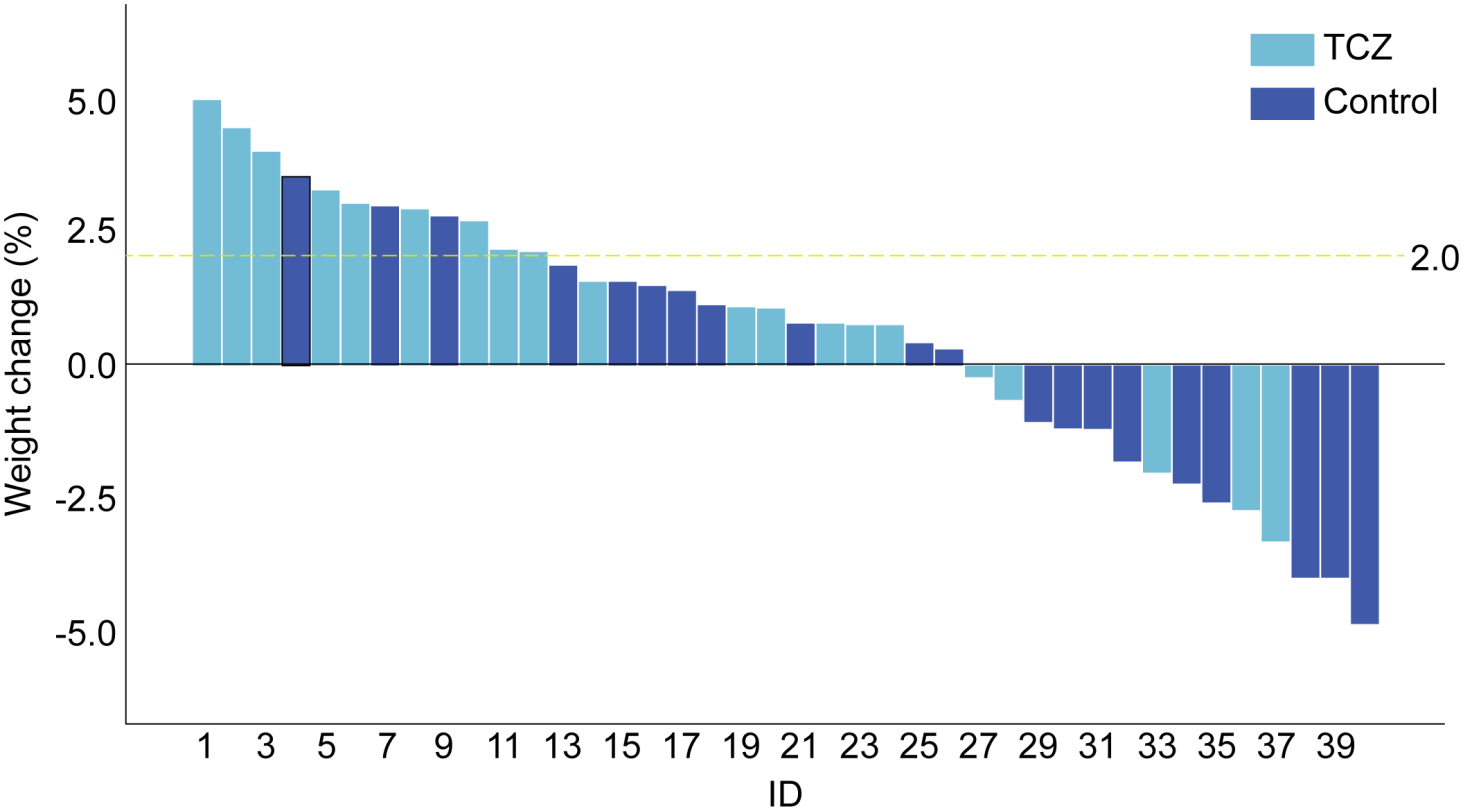
Weight change of patients on day 30. The 2% cut-off value reference to Fearon criteria.

**Figure 5:**
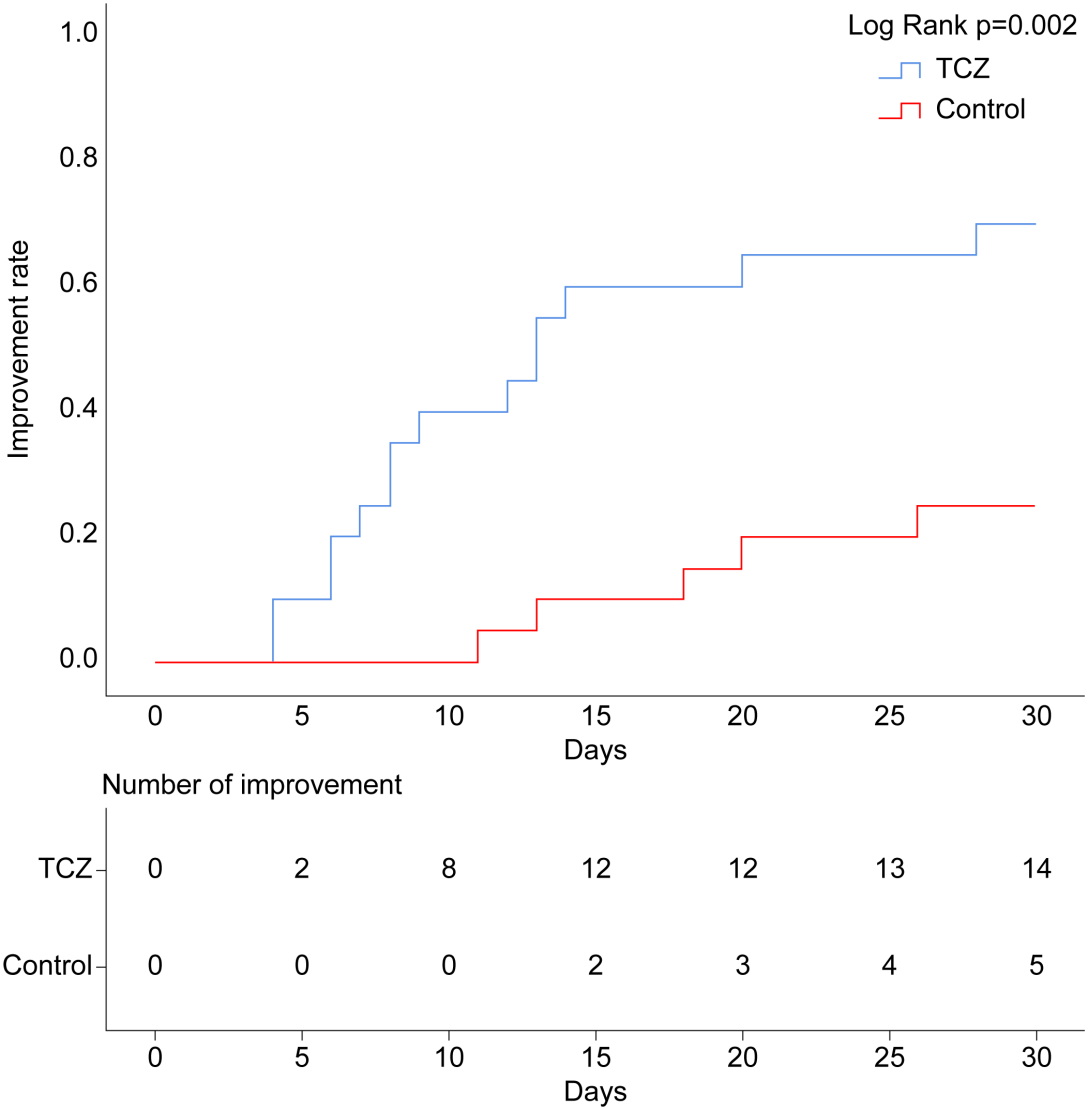
Cumulative occurrence curve for serum PAB and ALB improvement. Improvement: PAB >180 mg/L and ALB >35 g/L. ALB, albumin; PAB, prealbumin.

After TCZ treatment, PG-SGA scores significantly improved compared to baseline [12 (7–26) vs. 6.5 (3–24) points, p = 0.001], while the control group showed no significant improvement [14 (9–17) vs. 12.5 (4–18), p = 0.265]. Handgrip strength [21.9 (15.4–39.7) vs. 28.4 (15.2–47.9), p = 0.043] and 6-m walk speed [0.9 (0.6–1.1) vs. 1.2 (0.7–1.3), p = 0.001] also significantly increased in the TCZ group compared with pre-treatment levels (**Figure 2**). There was no significant difference in body weight between the two groups before and after treatment (p = 0.620 and p = 0.968, respectively) (**Table 2**). Additionally, the rate of weight gain did not differ significantly between the two groups at the 1-month follow-up (75% vs. 55%, p = 0.185). However, a higher percentage of patients in the TCZ group experienced weight gain exceeding 2% of their body weight compared to the control group (45% vs. 15%, p = 0.038) (**Figure 4**). The PAB and ALB levels significantly improved within 1 month in the TCZ group (70% vs. 25%, p = 0.004), and this recovery occurred more rapidly (**Figure 5**).

### 3.4 Safety of TCZ

The incidence of infection during treatment was comparable between the groups (10% vs. 15%, p = 0.633). In the TCZ group, infections included one case of respiratory infection and one case of intestinal infection, while in the control group, there were two cases of respiratory infections and one case of perianal infection. All infections resolved following sensitive antibiotic treatment. Throughout the follow-up period, although the ICU admission rate was lower in the TCZ group (10% vs. 25%), the difference was not statistically significant (p = 0.405) (**Table 2**). Two patients in the TCZ group were admitted to the ICU: one due to airway obstruction and one due to recurrent epileptic episodes, both attributed to the underlying tumor itself.

Within 1 month, assessment based on RECIST 1.1 criteria revealed no significant difference in tumor response between the two groups (p = 0.439). However, the TCZ group exhibited a higher rate of restarting antitumor therapy (including immunotherapy, targeted therapy, or chemotherapy) at follow-up compared to the control group (70% vs. 35%, p = 0.027) (**Table 2**). In the TCZ group, chemotherapy alone (n = 3), targeted therapy alone (n = 5), and combination therapy (n = 6) were administered, while the control group received chemotherapy alone (n = 2), immunotherapy alone (n = 2), targeted therapy alone (n = 2), and combination therapy (n = 1). In summary, TCZ administration did not increase the incidence of infections, and no adverse effects on oncological therapy were observed.

## 4 Discussion

In the present retrospective study comprising 40 cases of cachexia with systemic hyperinflammation, we conducted a comparative analysis of alterations in clinical parameters and laboratory biomarkers between the TCZ and the control groups. We found that TCZ suppressed inflammation well and significantly improved clinical symptoms and nutritional status in this population.

In this study, patients typically displayed elevated NLR and CRP levels, decreased PAB and ALB levels, along with symptoms such as low-grade fever, malaise, and anemia. Previous studies have revealed that IL6-mediated inflammatory pathways are associated with cachexia. The classical signaling pathway of IL-6-mediated inflammation orchestrates target organ proliferation, regeneration, and acute-phase responses by recruiting two gp130 subunits and eliciting downstream Janus kinase/signal transducer and activator of transcription and extracellular signal-regulated kinase signaling through interactions between IL-6 and IL-6R (17, 18). In cachexic stroma marked by a concurrent hyperinflammatory state, there is excessive proliferation and activation of target tissues, along with a prolonged expression of acute-phase proteins (19), inducing a hypermetabolic state within the organism. This triggers the recruitment of soluble IL-6R signaling, exacerbates inflammation, and creates a vicious cycle. Hence, it is crucial to underscore the significance of monitoring a patient’s inflammatory state as a pivotal aspect of early recognition of cachexia (20, 21). The lack of a well-defined threshold for hyperinflammation in cachexia, combined with the inclusion of various tumor types in our study, further complicated the identification of an appropriate cutoff. A CRP level greater than 10 mg/L, as one of the criteria in the modified Glasgow Prognostic Score, has been associated with poor prognosis across various malignancies (22). Notably, the baseline CRP levels in our patient cohort were significantly elevated above this tumor-specific threshold, indicating a hyperinflammatory state.

Regarding clinical outcomes, individuals in the TCZ group demonstrated a higher rate of symptom remission compared to the corresponding in the control group, including low-grade fever and anorexia, which are common manifestations of cachexia. TCZ treatment resulted in a notable reduction in the NLR and CRP levels, accompanied by an increase in the Hb levels compared to the control group. Furthermore, We systematically assessed the effect of TCZ on patients’ nutritional status. Compared with the control group, patients treated with TCZ exhibited significantly superior recovery in nutritional levels. This superiority was evident in the markedly elevated PAB and ALB levels in the TCZ group, higher rate of improvement, faster recovery timeframe during the 30-day follow-up period, higher incidence of weight gain exceeding 2% within 30 days, and more substantial improvement in the PG-SGA scores, all of which demonstrated statistically significant differences. TCZ shown potent anti-inflammatory effects and short-term efficacy.

Indeed, recent studies have shown that ALB was actually an inflammatory parameter rather than a serum nutritional indicator and that PAB is affected by a combination of nutritional status and inflammatory response (23, 24), so that restoration of serum PAB and ALB levels implied a decrease in inflammation and an improvement in nutrition in the organism. To avoid the controversy of the side effect of glucocorticoid sodium water storage on body weight, we assessed the patients’ muscle strength and physical performance using handgrip strength and 6-m walk speed, confirming the positive effect of TCZ on muscle function. After treatment with TCZ, our follow-up within one month indicated improvements in the patient’s anorexia symptoms, a decrease in inflammation, and increases in both grip strength and walk speed. Consequently, based on Fearon’s criteria, we concluded that the patient’s cachexia had improved compared to the previous period.

In fact, as early as 1993, it was claimed that IL-6 monoclonal antibody or IL-6 receptor antagonist treatment significantly inhibited the development of cancer cachexia in homozygous mice (25). Studies in rodents have elucidated a close association between the IL-6/IL-6R/STAT-3 cascade and muscle degradation during cachexia progression (26, 27). Moreover, prolonged IL-6 activation induces anemia and elevates plasma levels of CRP and immunoglobulins (28), potentially affecting the intestinal microbiota. In severe cachexia, sustained activation may also increase intestinal permeability, thereby affecting nutrient absorption and compromising the systemic nutritional status (29). These theories may elucidate the improvement in appetite and nutritional status observed after TCZ treatment in our study, and why treatment with corticosteroids alone showed less effectiveness.

Given its potent immunosuppressive nature, the potential to exacerbate or cause new infections after TCZ treatment remains the primary limitation that hinders its widespread clinical application. Individuals with congenital IL-6R deficiency experience recurrent bacterial infections (30), prompting concerns among clinical experts that IL-6R inhibition may compromise the body’s ability to resist pathogens, leading to severe infections, which is a high risk for patients with cachexia. However, existing data suggest that the annual incidence of consecutive infectious adverse events among patients treated with IL-6R inhibitors ranges from 4.7 to 9.09 per 100 patients, with serious infectious events rarely occurring (31). This trend may be partly attributed to regular clinic visits, early detection, appropriate management of infections, and the discontinuation of IL-6R inhibitor therapy upon suspicion of infection. In this study, TCZ was administered in a single session. The incidence of concurrent infections after treatment was similar between the two groups, and administration did not result in an increased rate of ICU admission.

In addition, some physicians have expressed concerns regarding the potential of IL-6 inhibition to induce tumor hyperprogression; however, there is no clear evidence to support this view. Conversely, combining CTLA-4 inhibitors with IL-6 inhibitors enhances the tumor microenvironment in malignant melanoma, leading to a significant reduction in tumor size and improved survival in mice (32). Ongoing human clinical studies (NCT04940299) are expected to provide conclusive results. In our study, no significant difference in tumor response was observed between the two groups, possibly owing to the small sample size and tumor type heterogeneity. We observed that patients in the TCZ group showed a greater chance of restarting antitumor therapy, suggesting a potentially positive role for TCZ in the treatment of tumors in this patient subset. Notably, no other serious adverse reactions associated with TCZ were observed.

This treatment approach was exploratory in nature; therefore, our study only included 20 patients after screening, resulting in a limited sample size. Moreover, we mainly analyzed cachexia subtypes with comorbid systemic inflammation based on the pharmacological mechanism of TCZ. Thus, discretion is required to determine whether the therapeutic dosage and frequency can be generalized to all patients with cachexia. Indeed, the optimal dose and administration schedule of TCZ for cachexia, as well as the long-term safety of its use, remain unclear. Therefore, careful assessment of patients is necessary. We look forward to more research in the future to address these issues.

There is ongoing debate regarding the use of serum IL-6 levels as a biomarker for treatment. Related studies have yielded contradictory results. one study found that rheumatoid arthritis patients with low IL-6 levels experienced better outcomes with TCZ (33), while another study reported the opposite (34). Consequently, the search for effective biomarkers in TCZ treatment represents a promising direction for future research. Moreover, it is widely acknowledged that cachexia is primarily associated with sarcopenia (35). Due to incomplete imaging, we were unable to include muscle data in our study. We anticipate that future large-scale prospective studies will address this gap.

## 5 Conclusions

In this exploratory study, the combination of TCZ and corticosteroids demonstrated potential in alleviating symptoms and improving nutritional status in cancer patients experiencing cachexia and systemic hyperinflammation. However, further research is required to determine its effectiveness as a targeted treatment for cancer cachexia.

## Data Availability

The raw data supporting the conclusions of this article will be made available by the authors, without undue reservation.
